# Synthesis and Characterization of Magnetic Molecularly Imprinted Polymer for the Enrichment of Ofloxacin Enantiomers in Fish Samples

**DOI:** 10.3390/molecules21070915

**Published:** 2016-07-14

**Authors:** Yan-Fei Wang, Huo-Xi Jin, Yang-Guang Wang, Li-Ye Yang, Xiao-Kun OuYang, Wei-Jian Wu

**Affiliations:** School of Food and Pharmacy, Zhejiang Ocean University, Zhoushan 316022, China; wyanfei1990@163.com (Y.-F.W.); jinhuoxi@163.com (H.-X.J.); liyey@zjou.edu.cn (L.-Y.Y.); weijain1955@hotmail.com (W.-J.W.)

**Keywords:** magnetic solid-phase extraction, ofloxacin enantiomers, magnetic molecularly imprinted polymers, chiral HPLC

## Abstract

A new method for the isolation and enrichment of ofloxacin enantiomers from fish samples was developed using magnetic molecularly imprinted polymers (MMIPs). These polymers can be easily collected and rapidly separated using an external magnetic field, and also exhibit a high specific recognition for ofloxacin enantiomers. The preparation of amino-functionalized MMIPs was carried out via suspension polymerization and a ring-opening reaction using *rac*-ofloxacin as a template, ethylenediamine as an active group, glycidyl methacrylate and methyl methacrylate as functional monomers, divinylbenzene as a cross-linker, and Fe_3_O_4_ nanoparticles as magnetic cores. The characteristics of the MMIPs were assessed using transmission electron microscopy (TEM), X-ray powder diffraction (XRD), Fourier-transform infrared spectroscopy (FT-IR), and vibrating sample magnetometer (VSM) measurements. Furthermore, the adsorption properties were determined using Langmuir and Freundlich isotherm models. The conditions for use of these MMIPs as magnetic solid-phase extraction (MSPE) sorbents, including pH, adsorption time, desorption time, and eluent, were investigated in detail. An extraction method using MMIPs coupled with high performance liquid chromatography (HPLC) was developed for the determination of ofloxacin enantiomers in fish samples. The limits of quantitation (LOQ) for the developed method were 0.059 and 0.067 μg∙mL^−1^ for levofloxacin and dextrofloxacin, respectively. The recovery of ofloxacin enantiomers ranged from 79.2% ± 5.6% to 84.4% ± 4.6% and ofloxacin enantiomers had good linear relationships within the concentration range of 0.25–5.0 μg∙mL^−1^ (R^2^ > 0.999). The obtained results demonstrate that MSPE-HPLC is a promising approach for preconcentration, purification, and simultaneous separation of ofloxacin enantiomers in biomatrix samples.

## 1. Introduction

In recent years, the differences in the pharmacology and pharmokinetics of enantiomers of chiral drugs have received increasing attention [1,2]. Ofloxacin (OFL), a third-generation quinolone [3], is a fully synthetic antimicrobial agent widely used in human and veterinary medicine [4,5]. Monitoring OFL enantiomer residues (*S*-(−)-OFL and *R*-(+)-OFL) in fish samples and other animal products for human consumption is of significant interest.

Recently, high performance liquid chromatography (HPLC) has become one of the most commonly used methods to separate chiral enantiomers [6,7,8,9]. However, interference from complex biological matrices and trace amounts of analytes makes it difficult to determine chiral enantiomers in biomatrix samples directly. Therefore, an efficient and selective pretreatment process is of particular importance. Traditional pretreatment methods include liquid–liquid extraction (LLE) [10,11], solid-phase extraction (SPE) [12], and micro-solid-phase extraction (μ-SPE) [13,14], which are laborious, time consuming, and use organic solvents. Simple, efficient, and fast pretreatment strategies using molecular imprinting techniques [15,16] or magnetic solid-phase extraction (MSPE) [17,18,19,20] have received more attention. The MSPE technique combines molecular imprinting techniques [21,22,23] and magnetic nanoparticles as a method for the enrichment and separation of analytes from complex matrices. Magnetic molecularly imprinted polymers (MMIPs) are used as an adsorbent for analytes in solution to achieve rapid separation under a magnetic field. Ofloxacin is a high-value, synthetic drug, and as molecularly imprinted polymers have been successfully proposed for large-scale engineering applications in recent years [24,25], novel ofloxacin-imprinted polymers could potentially be used for large-scale environmental purposes as well.

In this study, new amino-functionalized MMIPs as MSPE sorbents were prepared for the extraction of OFL enantiomers in fish samples. The obtained MMIPs were characterized by transmission electron microscopy (TEM), X-ray powder diffraction (XRD), vibrating sample magnetometer (VSM) measurements, and Fourier-transform infrared spectroscopy (FT-IR). Moreover, the adsorption properties and extraction conditions were investigated. As expected, a method based on MSPE coupled with chiral-HPLC analysis was successfully optimized for the separation and determination of OFL enantiomers in fish samples. This proposed technique was shown to be a reliable and effective analytical method for determination of trace amounts of chiral drugs in biomatrix samples. Moreover, enantiopure templates can also be used for the preparation of imprinted polymers with enantiodiscriminative features that could be successfully applied for enantioseparation and drug delivery in the future [26,27].

## 2. Results and Discussion

### 2.1. Synthesis and Characterization of MMIPs

**Figure 1:**
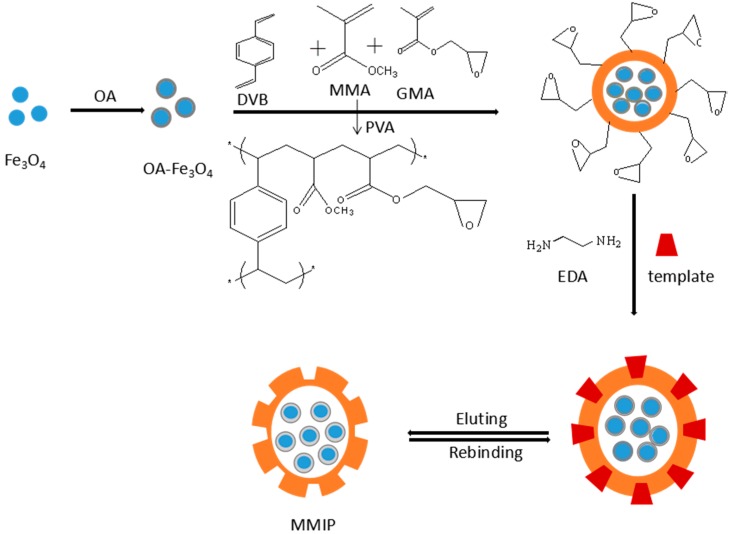
MMIPs preparation procedure (OA is oleic acid, DVB is divinylbenzene, MMA is methyl methacrylate, GMA is glycidyl methacrylate, PVA is poly(vinyl alcohol) 1788, and EDA is ethylenediamine).

In this work, MMIPs were synthesized by suspension polymerization. A schematic of the MMIP preparation process is shown in Figure 1. First, superparamagnetic Fe_3_O_4_ nanoparticles were prepared by a coprecipitation method. As these particles are hydrophilic, they cannot be effectively combined with functional monomers. Therefore, the Fe_3_O_4_ particles were coated with oleic acid (OA) to modify the properties of the hydrophobic Fe_3_O_4_ nanoparticles. Using glycidyl methacrylate (GMA) and methyl methacrylate (MMA) as functional monomers, divinylbenzene (DVB) as a cross-linker, poly(vinyl alcohol) (PVA) as a dispersant, and benzoyl peroxide (BPO) as an initiator, magnetic polymers containing epoxy groups were successfully synthesized. In the presence of ethylenediamine (EDA) and OFL, a ring-opening reaction occurred, and MMIPs with included template molecules were prepared. Finally, MMIPs with surface binding sites were achieved by removal of the templates.

**Figure 2:**
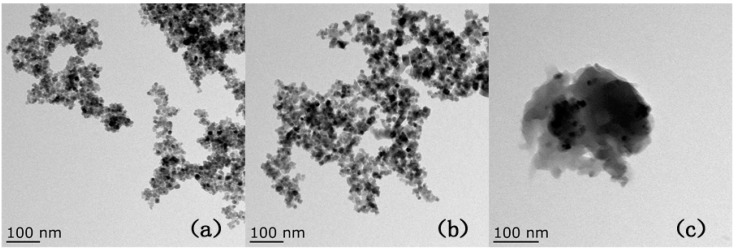
TEM images of Fe_3_O_4_ (**a**); OA-Fe_3_O_4_ (**b**); and MMIPs (**c**).

The morphological features of Fe_3_O_4_ (a), OA-Fe_3_O_4_ (b), and the MMIPs (c) were observed by TEM (Figure 2). These images revealed that the Fe_3_O_4_ nanoparticles had irregular spherical shapes with diameters of about 20 nm. Some of the magnetic nanoparticles were aggregated with larger particles (Figure 2a). Following OA modification (Figure 2b), the dispersity of the particles improved and aggregation of the particles was significantly reduced. The presence of OA on the Fe_3_O_4_ surface may have increased steric hindrance, thus preventing effective aggregation. The obtained MMIPs nanoparticles exhibited a well-defined core-shell configuration with a diameter of about 200 nm, as shown in Figure 2c. The grey areas are polymer layers, whereas the black areas are the cores, which contain many magnetic nanoparticles.

**Figure 3:**
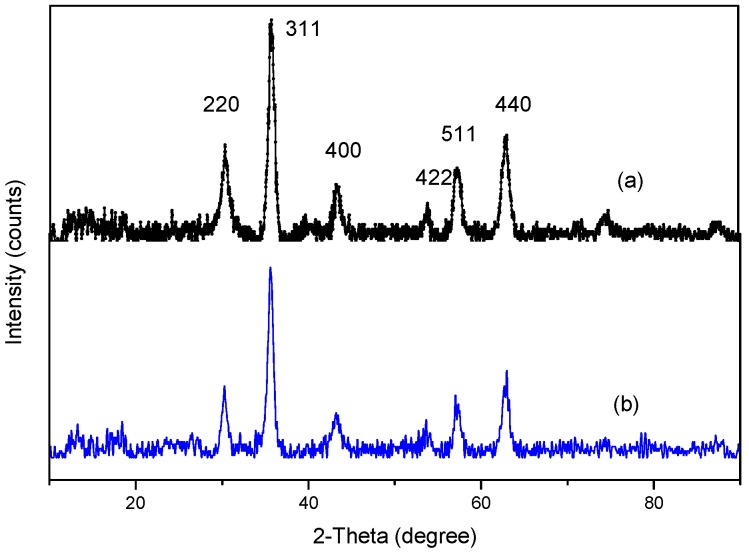
XRD patterns of Fe_3_O_4_ (**a**) and MMIPs (**b**).

The XRD patterns of Fe_3_O_4_ (a) and the MMIPs (b) were obtained, as shown in Figure 3. Both XRD spectra show six peaks that are characteristic of Fe_3_O_4_ at 2 θ = 30.4°, 35.8°, 43.3°, 53.9°, 57.3°, and 63.1°, which correspond to the (220), (311), (400), (422), (511), and (440) indices, respectively. This result reveals that the crystal structure of Fe_3_O_4_ remained stable during the polymerization process and Fe_3_O_4_ was incorporated into the MMIPs. The intensity of the characteristic peaks for MMIPs is lower than that for Fe_3_O_4_, which may be due to blocking of magnetic expression by the thick polymer layers on the MMIPs’ surface. This is consistent with MMIPs having a larger diameter than Fe_3_O_4_, as indicated in the TEM results.

**Figure 4:**
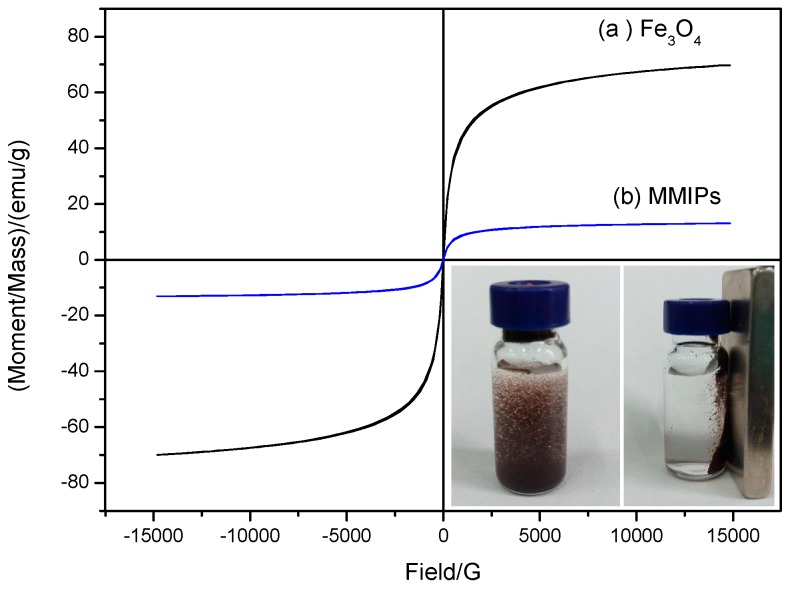
Magnetic hysteresis loops of Fe_3_O_4_ (**a**) and MMIPs (**b**).

Figure 4 shows the magnetic hysteresis loop analysis of Fe_3_O_4_ (a) and the MMIPs (b). There was no apparent hysteresis in either curve, suggesting that Fe_3_O_4_ and MMIPs were superparamagnetic. The saturation magnetization of Fe_3_O_4_ and MMIPs was 69.733 and 13.046 emu g^−1^, respectively. As shown in the inset photograph in Figure 4, the dispersed MMIPs were easily attracted to the wall of a vial under an external magnetic field. This result showed that MMIPs exhibit an adequate magnetic response to undergo magnetic separation.

**Figure 5:**
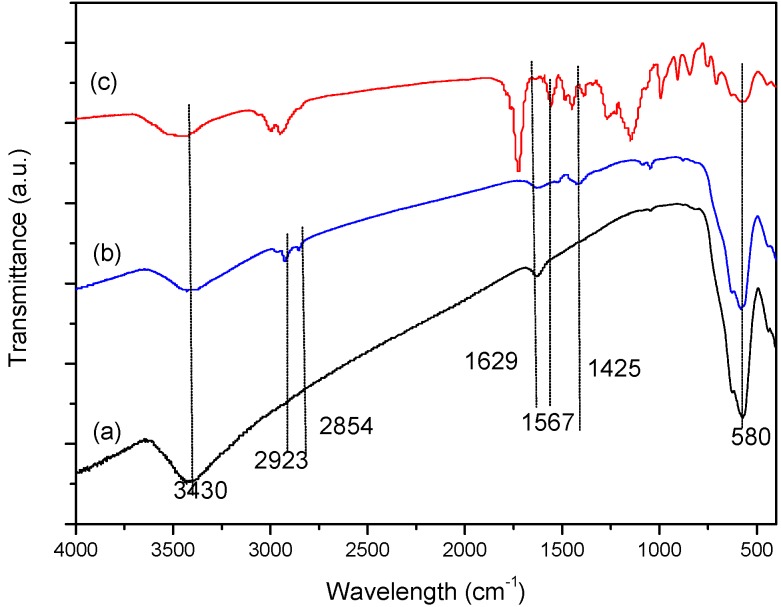
FT-IR spectra of Fe_3_O_4_ (**a**); OA-Fe_3_O_4_ (**b**); and MMIPs (**c**).

The FT-IR spectra of Fe_3_O_4_ (a), OA-Fe_3_O_4_ (b), and the MMIPs (c) are shown in Figure 5. The main functional groups of the predicted structures can be inferred from the infrared adsorption peaks. The adsorption peak at 580 cm^−1^ in the spectra of Fe_3_O_4_, OA-Fe_3_O_4_, and the MMIPs corresponds to the Fe-O bond of Fe_3_O_4_ particles. After OA modification (Figure 5b), new characteristic peaks located at 2854, 2923, 1425, and 1629 cm^−1^ arose, which correspond to the stretching vibrations of -CH_3_ and -CH_2_-, and the bending vibration of carboxylate. These peaks are consistent with successful coating of OA onto Fe_3_O_4_. The new peak at 1567 cm^−1^ observed for the MMIPs (Figure 5c) corresponds to the characteristic adsorption of -N-H, which indicates that the amino-functionalized MMIPs were successfully synthesized.

### 2.2. Adsorption Isotherms

**Figure 6:**
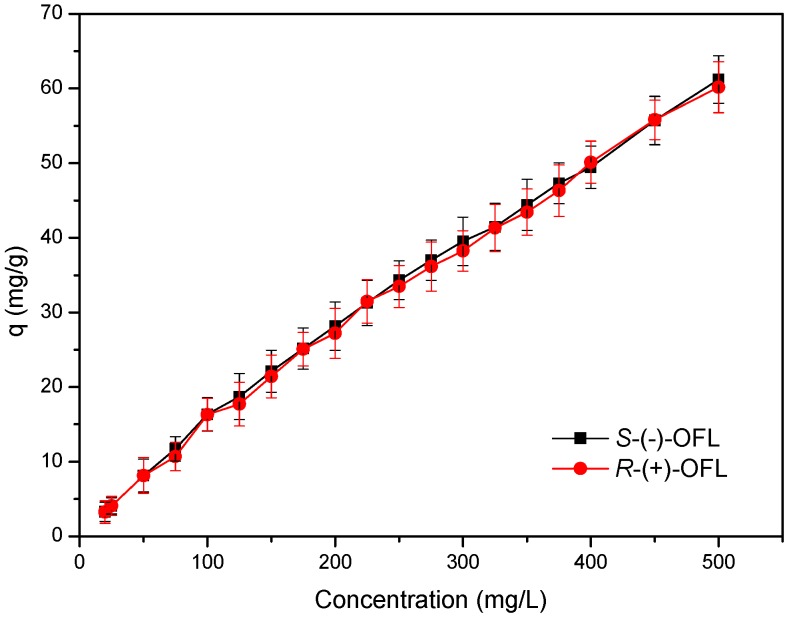
Adsorption isotherms for adsorption of OFL onto MMIPs.

The adsorption isotherms of the MMIPs were investigated by dispersing the adsorbents in OFL solutions (20–1000 mg∙L^−1^) and shaking for 30 min. After separation with a magnet, the supernatants were analyzed by HPLC. Figure 6 shows the adsorption isotherms of OFL on the MMIPs. The results indicated that the adsorption capacity of the MMIPs for *S*-(−)-OFL and *R*-(+)-OFL increased linearly with increasing initial concentration of OFL, with high adsorption capacities for OFL exhibited. A *t*-test of the q values of *S*-(−)-OFL and *R*-(+)-OFL indicated that there was no significant difference between the adsorption of *S*-(−)-OFL and *R*-(+)-OFL on the MMIPs that used *rac*-OFL as the template (*p* > 0.05). Moreover, when using MMIPs as solid adsorbents, the abundant surface recognition sites resulted in increased binding capacity, which should allow the enrichment of trace OFL enantiomers from complex systems. To further estimate the adsorption properties of the MMIPs, two classical isotherm models, Langmuir and Freundlich, were selected to fit the experimental data. The Langmuir equation can be used to describe monolayer adsorption, whereas the Freundlich equation can be used to describe monolayer adsorption, as well as multilayer adsorption. Table 1 lists the parameters obtained using the Freundlich and Langmuir isotherm models, as well as the correlation coefficients (R^2^) for the adsorption data. For both *S*-(−)-OFL and *R*-(+)-OFL, the R^2^ value for the Freundlich model was somewhat higher than that for the Langmuir model. Therefore, the adsorption amounts of OFL on the MMIPs were fitted to the Freundlich isotherm model. Thus, multilayer OFL coverage on the surface of the MMIPs was verified.

### 2.3. Optimization of MSPE Conditions

#### 2.3.1. Effect of pH Value

**Figure 7:**
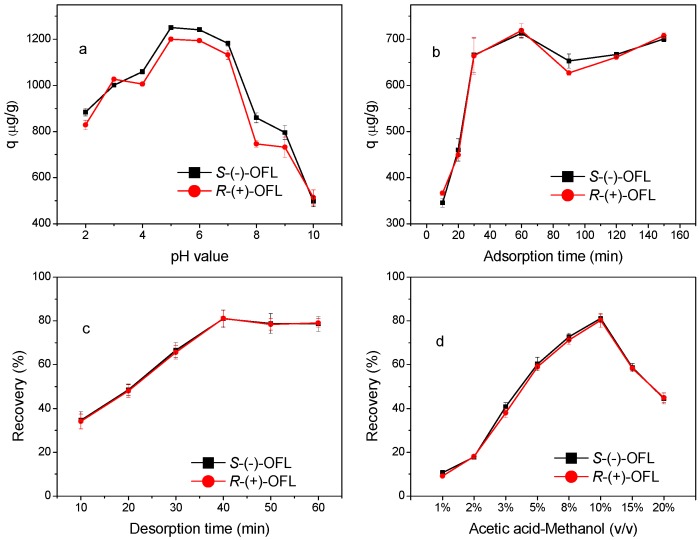
Optimisation of extraction conditions: Effect of pH on OFL adsorption onto MMIPs (adsorption time of 30 min) (**a**), effect of adsorption time on OFL adsorption onto MMIPs (pH 5) (**b**), effect of desorption time on the recovery of OFL (**c**), effect of elute solvent on the recovery of OFL (**d**).

The effect of the solution pH (2.0–10.0) on the adsorption of OFL by the MMIPs was investigated using 20.0 μg∙mL^−1^ OFL solutions, as shown in Figure 7a. The OFL adsorption capacity was highly dependent on pH. The q values of *S*-(−)-OFL and *R*-(+)-OFL gradually increased as the pH increased from 2.0 to 5.0. Moreover, pH values of 5.0 and 6.0 were optimal for OFL adsorption. As the solution pH increased from 6.0 to 10.0, the q values sharply decreased. A t test of the q values of *S*-(−)-OFL and *R*-(+)-OFL showed that there was no significant difference (*p* > 0.05) between the adsorption of *S*-(−)-OFL and *R*-(+)-OFL on the MMIPs using *rac*-OFL as the template.

The dependence of OFL adsorption on pH can be explained from the perspective of surface chemistry and the ionization state of OFL in the aqueous phase. The primary driving force for binding between OFL and the MMIPs is hydrogen bonding, which is strongly related to the solution pH. In the present work, the ionization states of OFL (pK_a1_ = 5.77, pK_a2_ = 8.44) [28] and the amino groups on MMIPs are significantly affected by the solution pH. When the pH is low (pH < 5.0), the amino groups on both the surface of the MMIPs and OFL are protonated and in an ionic state. There is electrostatic repulsion between the MMIPs and OFL and the formation of hydrogen bonds is difficult, leading to poor OFL adsorption efficiency. When the pH is 5.0–6.0, the amino groups on OFL and the MMIPs and the carboxyl groups on OFL are all in the molecular state and hydrogen bonds (-O-H···N, -C=O···H, and -N-H···N) can easily form; therefore, the highest adsorption efficiencies were achieved at pH values of 5.0 and 6.0. At higher solution pH values (pH > 6.0), the carboxyl groups on OFL are gradually deprotonated to an ionic state, resulting in a decline in the adsorption amounts. Thus, pH = 5.0 was selected for all subsequent adsorption experiments.

#### 2.3.2. Adsorption Time

To monitor the adsorption kinetics of the MMIPs, the effect of adsorption time was investigated by varying the shaking time (0–150 min), as shown in the kinetic curves in Figure 7b. The adsorption capacities of *S*-(−)-OFL and *R*-(+)-OFL increased with increasing adsorption time and reached adsorption equilibrium at 30 min; thus, 30 min was chosen as the optimal extraction time. This fast adsorption could be due to a large number of active sites on the surface imprinting cavities of the MMIPs, which results in faster diffusion from the solution to the active sites. A t test of the q values of *S*-(−)-OFL and *R*-(+)-OFL indicated that there is no significant difference between the adsorption of *S*-(−)-OFL and *R*-(+)-OFL on MMIPs using *rac*-OFL as the template with increasing time (*p* > 0.05). The adsorption kinetic data obtained from batch experiments were analyzed using pseudo-first-order and pseudo-second-order equations. The equations and the calculated k and q values are listed in Table 2. The results indicated that the pseudo-second-order model better described the adsorption process for OFL onto the MMIPs. The calculated equilibrium adsorption capacities (q_e,c_) of *S*-(−)-OFL and *R*-(+)-OFL from the pseudo-second-order model are closer to the experimental q_e_ values.

#### 2.3.3. Desorption Conditions

The desorption time and elution solvent, which are the main parameters for the desorption process, were optimized. Experiments with desorption times in the range of 10–60 min were carried out, as shown in Figure 7c. The recoveries of OFL increased with increasing desorption time from 10 to 40 min. Therefore, 40 min was selected as the optimal desorption time.

Various proportions of acetic acid–methanol were investigated to obtain satisfactory recoveries. As shown in Figure 7d, 10% acetic acid–methanol (*v*/*v*) was found to be the most effective eluent for OFL. The recoveries increased as the proportion of acetic acid increased from 1% to 10%. However, the MMIPs may be destroyed at higher concentrations of acetic acid [28]. Thus, 5 mL of 10% acetic acid-methanol was adopted for the desorption of OFL.

### 2.4. Reusability of MMIPs

**Figure 8:**
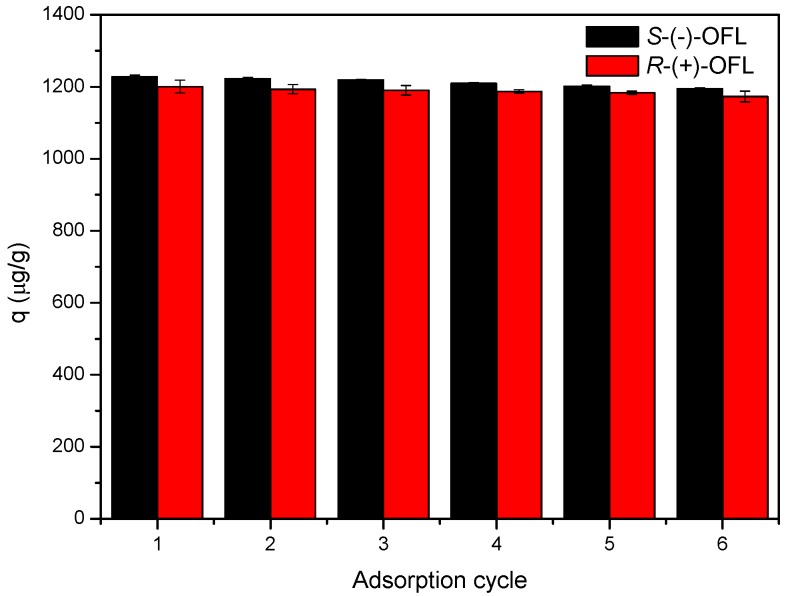
Adsorption capacity of OFL on MMIPs over six adsorption cycles.

The reusability of the MMIPs was evaluated by determining the adsorption capacity of the MMIPs after regeneration. The MMIPs could be regenerated by treatment with 10% acetic acid–methanol (*v*/*v*) for 1 h, and then reused for the adsorption of OFL. As shown in Figure 8, the MMIPs could be reused at least six times for the extraction of *S*-(−)-OFL and *R*-(+)-OFL with a slight decrease in the adsorption capacity (2.3% and 2.7%, respectively). This result implies that the MMIPs are stable and can be recycled.

### 2.5. Imprinting Effects of MMIPs and MNIPs on OFL Adsorption

As shown in Table 3, the adsorption capacities of *S*-(−)-OFL and *R*-(+)-OFL on MMIPs were much higher than those on magnetic non-imprinted polymers (MNIPs), which indicated that the MMIPs had high selectivity for OFL. Moreover, the α values were greater than 1, indicating that the MMIPs had a higher affinity towards the target molecules than the MNIPs. The selectivity of the MMIPs mainly depends on the binding between the MMIPs and the target molecules, with the binding ability related to the similarities between the functional groups, size and shape of the template, and the target molecules. The results illustrated the success of the imprinting process. After the removal of the template, the imprinting sites on the surface of the MMIPs were accessible to the OFL target molecules.

### 2.6. Application of MMIPs to Biomatrix Samples

A series of experiments were carried out to evaluate the proposed method. The linear range, correlation coefficient (R^2^), limit of detection (LOD), and limit of quantitation (LOQ) were determined and used to validate the analytical methodology in this work. Calibration curves were built using standard solutions of OFL enantiomers in the concentration range of 0.25–5 μg∙mL^−1^. The regression equations were y = 108.25x − 4.183 (R^2^ = 0.9996) and y = 118.16x − 7.482 (R^2^ = 0.9998) for *S*-(−)-OFL and *R*-(+)-OFL, respectively. The LOD and LOQ values, defined as 3 and 10 times the signal-to-noise ratio, were 0.018 and 0.059 μg∙mL^−1^, and 0.020 and 0.067 μg∙mL^−1^ for *S*-(−)-OFL and *R*-(+)-OFL, respectively.

**Figure 9:**
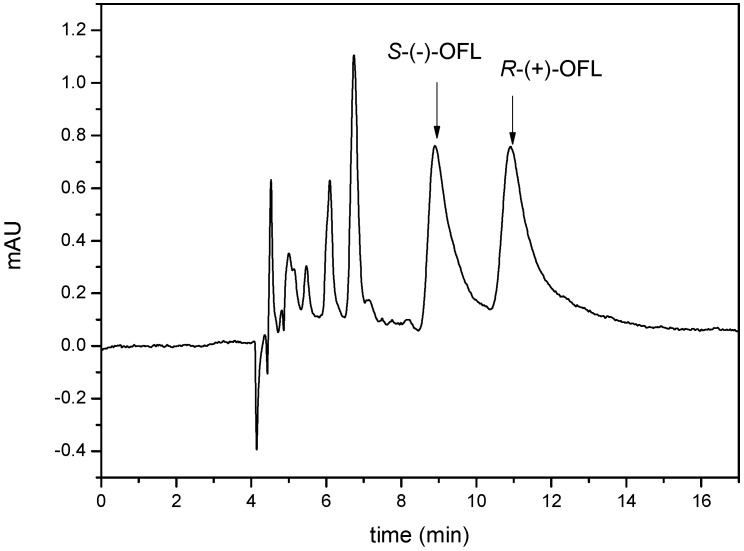
Chromatogram of a solution eluted from MMIPs with 10% acetic acid–methanol (*v*/*v*).

Under the optimized conditions, the MMIPs were applied to the analysis of OFL in fish samples. Fish samples spiked with 0.5, 2.5, and 5.0 μg∙g^−1^ of OFL were used to evaluate the repeatability, accuracy, and recovery of the MMIPs as a sorbent for the extraction process (Table 4). The intra-day precision was evaluated by measuring the recovery of OFL six times in one day, whereas the inter-day precision was investigated by analyzing the recovery of OFL on six consecutive days. The recoveries of the spiked fish samples for *S*-(−)-OFL and *R*-(+)-OFL ranged from 79.3% to 84.1% and 79.2% to 84.4%, respectively. Moreover, for *S*-(−)-OFL, the relative standard deviations (RSDs) of the intra- and inter-day recoveries ranged from 2.9% to 6.0% (*n* = 6), whereas for *R*-(+)-OFL, the RSDs of the intra- and inter-day recoveries ranged from 3.2% to 5.6% (*n* = 6). Figure 9 shows the chromatogram of the solution eluted from OFL-loaded MMIPs with 10% acetic acid-methanol. These results revealed that the use of the MMIPs as a MSPE sorbent coupled with chiral HPLC can be applied to the selective adsorption and determination of OFL enantiomers in fish samples.

## 3. Materials and Methods

### 3.1. Materials

Analytical-grade *rac*-ofloxacin (98.0%, OFL), iron chloride hexahydrate (FeCl_3_∙6H_2_O), iron chloride tetrahydrate (FeCl_2_∙4H_2_O), methyl methacrylate (MMA), benzoyl peroxide (BPO), glycidyl methacrylate (GMA), divinylbenzene (DVB), and poly(vinyl alcohol) 1788 (PVA) were purchased from Aladdin Chemical Reagent Co., Ltd. (Shanghai, China). Analytical-grade ammonia solution (NH_3_∙H_2_O), oleic acid (OA), ethylenediamine (EDA), sodium hydroxide (NaOH), methanol, hydrochloric acid (HCl), acetonitrile (ACN), acetic acid, and triethylamine were obtained from Sinopharm Chemical Reagent Co., Ltd. (Shanghai, China). The levofloxacin (*S*-(−)-OFL, >99.0%) and dextrofloxacin (*R*-(+)-OFL, >99.0%) standards were provided by Daicel Chiral Technologies Co., Ltd. (Shanghai, China). HPLC-grade ethanol and *n*-hexane were purchased from Oceanpak Alexative Chemical, Ltd. (Gothenburg, Sweden). Deionized water was supplied by a Milli-Q water purification system from Millipore (Molsheim, France). The fish samples (*Sciaenops ocellatus*) were acquired from local markets.

An HH-1 digital electronic thermostat water bath (Changzhou Guohua Electric Co., Ltd., Jiangsu, China), JJ-1 Precision Force electric mixer (Changzhou Guohua Electric Co., Ltd.), and FD-1E freeze dryer (Beijing Detianyou Science and Technology Development Co., Ltd., Beijing, China) were used.

### 3.2. Chromatographic Conditions

The HPLC analyses were performed using an Agilent 1200 system (Agilent, Santa Clara, CA, USA) equipped with a quaternary pump (G1311A), column thermostat (G1316A), degasser unit (G1322A), autosampler (G1329A), and diode-array detector (G1315D). A Chiralcel OD-H (250 mm × 4.6 μm, 5 μm; Daicel, Japan) column was used for separation. The mobile phase was an *n*-hexane–ethanol mixture (20:80, *v*/*v*) at a flow rate of 0.7 mL∙min^−1^. Acetic acid (0.2% in ethanol (*v*/*v*)) and triethylamine (0.2% in ethanol (*v*/*v*)) were used as additives. The detection wavelength was 294 nm, 20 μL of analyte was injected, and the column temperature was 25 °C [29]. The HPLC system was controlled and the data were analyzed using a computer equipped with ChemStation software (Rev.B.04.02, Agilent).

### 3.3. Preparation of Rac-Ofloxacin MMIPs

Magnetic Fe_3_O_4_ nanoparticles were synthesized by a coprecipitation method according to the reported procedure [30,31] with a minor modification. Briefly, FeCl_3_∙6H_2_O (2.4 g) and FeCl_2_∙4H_2_O (0.9 g) were dissolved in deionized water (80 mL), and this solution was purged with nitrogen for 30 min to displace oxygen. The mixture was stirred and heated to 75 °C, and then NH_3_∙H_2_O was added dropwise to adjust the pH to 9–10. After 10 min, 10 mL of OA was added into the mixture, which was reacted for a further 1 h, and then allowed to cool to room temperature. Finally, the obtained OA-Fe_3_O_4_ nanoparticles were washed with water and ethanol several times to remove excess NH_3_·H_2_O and OA.

The MMIPs were synthesized by suspension polymerization according to the literature procedure [32,33,34,35] with a minor modification. PVA (2.0 g) was dissolved in 200 mL of deionized water, followed by the addition of 4.3 mL of MMA, 0.5 mL of DVB, and 6.8 mL of GMA. Then, 1 g of OA-Fe_3_O_4_ was added to the above system under ultrasonication (KQ-250B ultrasonic cleaner, Kunshan Ultrasonic Instrument Co., Ltd., Jiangsu, China). Finally, 1.0 g of BPO in 20 mL of ethanol was added dropwise under vigorous stirring; nitrogen was bubbled into the reaction mixture throughout the procedure. The mixture was reacted at 80 °C for 2 h, and the product was isolated under a magnetic field and washed with water and ethanol. Exactly 2 g of obtained particles was dispersed into 50 mL of methanol containing 1.0 mmol of *rac*-OFL, and 10 mL of EDA was added dropwise under stirring. The reaction was then maintained at 80 °C for 8 h. Upon completion of the reaction, the nanoparticles were isolated with an external magnetic field and washed with water and methanol. Subsequently, the obtained particles were ultrasonically cleaned with 10% (*v*/*v*) acetic acid in methanol for 30 min until template molecules were no longer observed by HPLC. Finally, the MMIPs were dried for 32 h in a freeze dryer at −40 °C. In parallel, MNIPs were prepared using the same procedure without *rac*-OFL.

### 3.4. Characterization

TEM experiments were carried out using a transmission electron microscope (JEM-2100, JOEL, Tokyo, Japan). Structures were determined using a D8 Advance powder X-ray diffraction spectrometer (XRD, Bruker, Karisruhe, Germany) equipped with a copper anode generating Cu Kα radiation (λ = 0.154 nm). FT-IR characterization was performed with a Thermo Nicolet 6700 FT-IR spectrometer (Thermo Nicolet, Waltham, MA, USA). The magnetic properties were measured using a VSM (Lake Shore 7410, Lake Shore Cryotronics, Westerville, FL, USA).

### 3.5. Adsorption Studies

Batch adsorption studies were performed by mixing 20 mg of MMIPs with 5 mL of OFL solution at various concentrations ranging from 20 to 1000 mg∙L^−1^ in a 50 mL conical flask. Both 0.1 mol∙L^−1^ HCl and 0.1 mol∙L^−1^ NaOH solutions were used to adjust the pH to 5. The solution was shaken at 150 rpm in a thermostatic shaker for 30 min. After magnetic separation, the OFL concentration in the supernatant was measured by HPLC. All tests were conducted in triplicate. Using the OFL concentrations before and after adsorption, the equilibrium adsorption capacity of OFL was calculated using the following equation [36,37]:

(1) $$q=\frac{(C_{0}-C_{e})\text{V}}{m}$$

where q (μg∙g^−1^) is the adsorption capacity of OFL bound on MMIPs, C_0_ and C_e_ are the initial solution concentration and equilibrium concentration of OFL (μg∙mL^−1^), respectively, V is the volume of the solution (mL), and m is the adsorbent dosage (mg).

To further analyze the adsorption data, the Langmuir equation and Freundlich model were used to estimate the binding properties of the MMIPs. The Langmuir equation is expressed as follows [38]:

(2) $$\frac{C_{e}}{q}=\frac{C_{e}}{q_{m}}+\frac{1}{q_{m}K_{L}}$$

where q is the adsorption capacity at equilibrium (mg∙g^−1^), q_m_ is the apparent maximum adsorption capacity (mg∙g^−1^), C_e_ is the equilibrium concentration of OFL (mg∙L^−1^), and K_L_ is the Langmuir constant related to the affinity of the adsorption sites (L∙mg^−1^). The values of K_L_ and q_m_ can be calculated from the slope and intercept of the linear regression fit for a plot of 1/q versus 1/C_e_.

The Freundlich isotherm is represented as follows [39,40]:

(3) $$\text{lgq}={\text{lgK}}_{F}+\frac{{\text{lgC}}_{e}}{n}$$

where n and K_F_ are Freundlich constants that are related to the adsorption favorability and adsorption capacity, respectively.

The adsorption kinetic data were analyzed using a pseudo-first-order Equation (4) and a pseudo-second-order Equation (5), as follows [41]:

(4) $$\ln(q_{e}-q_{t})=\ln q_{e}-k_{1}t$$

(5) $$\frac{t}{q_{t}}=\frac{1}{k_{2}q_{e,c}{}^{2}}+\frac{t}{q_{e,c}}$$

where q_e_ and q_t_ are the adsorption amounts (mg∙g^−1^) of OFL bound to the MMIPs at equilibrium and at any time t, respectively, and k_1_ (min^−1^) and k_2_ (g∙mg^−1^∙min^−1^) are the pseudo-first-order rate constant and pseudo-second-order rate constant at equilibrium, respectively.

After adsorption, the MMIPs loaded with OFL were eluted with 1%, 2%, 3%, 5%, 8%, 10%, 15%, or 20% methanol–acetic acid (*v*/*v*). The supernatants were evaporated to dryness under a stream of nitrogen, and the residues were redissolved in the mobile phase. Then, 20 μL of the resulting solution was used for HPLC analysis. After elution, the MMIPs were dried for 32 h in a freeze dryer at −40 °C and reused for OFL adsorption.

### 3.6. Imprinting Effects of MMIPs and MNIPs on OFL Adsorption

To evaluate the imprinting effects of the MMIPs towards OFL, 20 mg of MMIPs and MNIPs were individually dispersed into 5 mL of 20 μg∙mL^−1^ OFL solution, which was then shaken for 30 min at room temperature. The concentrations of *S*-(−)-OFL and *R*-(+)-OFL in the supernatants were measured by HPLC. The adsorption amounts of OFL on MMIPs and MNIPs were then compared.

The molecular recognition characteristics of MMIPs were evaluated using the partition coefficients for OFL between the particles and solution. The partition coefficient *K* can be expressed as follows [42]:

(6) $$K=q/{\text{C}}_{e}$$

where q is the adsorption amount of the target molecule for MMIPs or MNIPs and C_e_ is the residual concentration of the target molecule in the solution after adsorption.

The imprinting effects of MMIPs and MNIPs towards OFL were evaluated using the imprinting factor α, which can be calculated as follows [43]:

(7) $$\alpha =\frac{K_{\text{MIP}}}{K_{\text{NIP}}}$$

where K_MIP_ and K_NIP_ are the partition coefficients of MMIPs and MNIPs, respectively, with the target molecules.

### 3.7. Separation Enrichment and Determination of OFL Enantiomers in Fish Samples

Individual fish were beheaded, boned, skinned, minced, crushed, and homogenized. Standard solutions of *rac*-OFL (20, 100, and 200 µL) with a concentration of 50 μg∙mL^−1^ were added to blank tissue samples (2.0 g) to obtain spiked levels of 0.5, 2.5, and 5 μg∙g^−1^. The spiked samples were placed into a 50 mL centrifuge tube and 5 mL of 1% acetic acid in acetonitrile was added. The samples were vortexed for 3 min, and then centrifuged at 6000 rpm for 5 min. The extraction process was repeated three times. The supernatant solution was collected in a 50 mL conical flask. Then, 50 mg of MMIPs was added and the solution was shaken at 150 rpm for 1 h at room temperature. After extraction, the MMIPs were isolated using an external magnetic field, and the supernatant was discarded from the flask. The MMIPs were washed with water and methanol, and then eluted with 5 mL of 10% acetic acid in methanol. The obtained supernatant was evaporated to dryness under a stream of nitrogen. The residue was redissolved in 1 mL of the mobile phase and filtered through a 0.22 μm membrane for further HPLC analysis.

## 4. Conclusions

The obtained MMIPs with specific recognition structures led to a high OFL binding capacity (121.4 mg∙g^−1^). The Freundlich model fitted the equilibrium data well, and the adsorption process could be described by a pseudo-second-order equation. The MMIPs were successfully applied to the separation and enrichment of OFL enantiomers from fish samples with acceptable recoveries ranging from 79.2% to 84.4%. Therefore, the MMIPs as an MSPE sorbent coupled with chiral-HPLC for determination of OFL enantiomers in fish samples is a promising alternative to traditional solid-phase extraction methods. Although the synthetic approach is laborious, complicated, and time-consuming, the present method reveals that MMIPs have potential application in the preconcentration and determination of enantiomers in complex samples. In addition to enrichment, imprinting technology also provides opportunities for the separation and isolation of enantiomers, and, in the future, enantiopure templates could be used to prepare molecularly imprinted polymers for application in enantioseparation and drug delivery.

## Figures and Tables

**Table 1:** Parameters from the isotherm models for the adsorption of OFL onto MMIPs.

Analytes	Freundlich Model			Langmuir Model		
	n	K_F_	R^2^	q_m_ (mg∙g^−1^)	K_L_ (L∙mg^−1^)	R^2^
*S*-(−)-OFL	1.279	0.833	0.9926	106.383	0.004	0.9507
*R*-(+)-OFL	1.274	0.824	0.9921	107.527	0.004	0.9523

**Table 2:** Kinetic equations and rate constants for the adsorption of OFL onto MMIPs.

Model	Analytes	Equations	k	q_e_	q_e,c_	R^2^
Pseudo-first-order model	*S*-(−)-OFL	ln(q_e_ − q_t_) = 0.551 − 0.144t	0.144	0.715	0.579	0.8458
	*R*-(+)-OFL	ln(q_e_ − q_t_) = 0.517 − 0.138t	0.138	0.712	0.598	0.8688
Pseudo-second-order model	*S*-(−)-OFL	t/q_t_ = 1.399t + 7.744	0.253	0.715	0.701	0.9929
	*R*-(+)-OFL	t/q_t_ = 1.404t + 7.945	0.248	0.712	0.708	0.9901

**Table 3:** Adsorption capacities, partition coefficients, and imprinting factors of OFL on MMIPs and MNIPs.

Analytes	q_MIP_ (μg∙g^−1^)	q_NIP_ (μg∙g^−1^)	K_MIP_ (mL∙g^−1^)	K_NIP_ (mL∙g^−1^)	α
*S*-(−)-OFL	1455.83	821.90	69.72	24.50	2.85
*R*-(+)-OFL	1449.15	824.00	68.95	24.59	2.80

**Table 4:** Recoveries and RSDs of OFL on MMIPs for spiked fish samples (*n* = 6).

Analytes	OFL Added (μg∙g^−1^)	Recovery (%)	Precision (RSD%)	
			Intra-Day	Inter-Day
*S*-(−)-OFL	0.25	79.3	3.5	6.0
	1.25	83.7	2.9	3.4
	2.5	84.1	3.9	4.2
*R*-(+)-OFL	0.25	79.2	3.6	5.6
	1.25	83.9	3.2	3.5
	2.5	84.4	4.0	4.6
